# Supplementary material for: Identifying Prenatal and Postnatal Determinants of Infant Growth: A Structural Equation Modelling Based Cohort Analysis
Source: Int J Environ Res Public Health. 2021 Sep 29;18(19):10265. doi: 10.3390/ijerph181910265 (PMC8507693; doi:10.3390/ijerph181910265)
Supplement: Supplementary file 1 [file ijerph-18-10265-s001.zip › ijerph-1390561-supplementary.pdf]

Supplementary Materials

Statistical Analyses

Based on existing literature, each variable which had a potential effect on infant size was individually tested through linear or logistic regression (results are located in Supplementary table S2). Using confirmatory factor analyses (CFA), the study outcome variable, infant size was modelled as a latent variable with two measured constructs, infant waist circumference and infant weight, both measured at the 12-month follow-up. CFA was also used to identify any clusters amongst significant variables however, no latent variables were apparent. Adopting a stepwise fashion, further regression analyses were carried out to identify any existing relationships between the significant variables and infant size. This process enabled an exploratory SEM model to be created (supplementary figure S1).

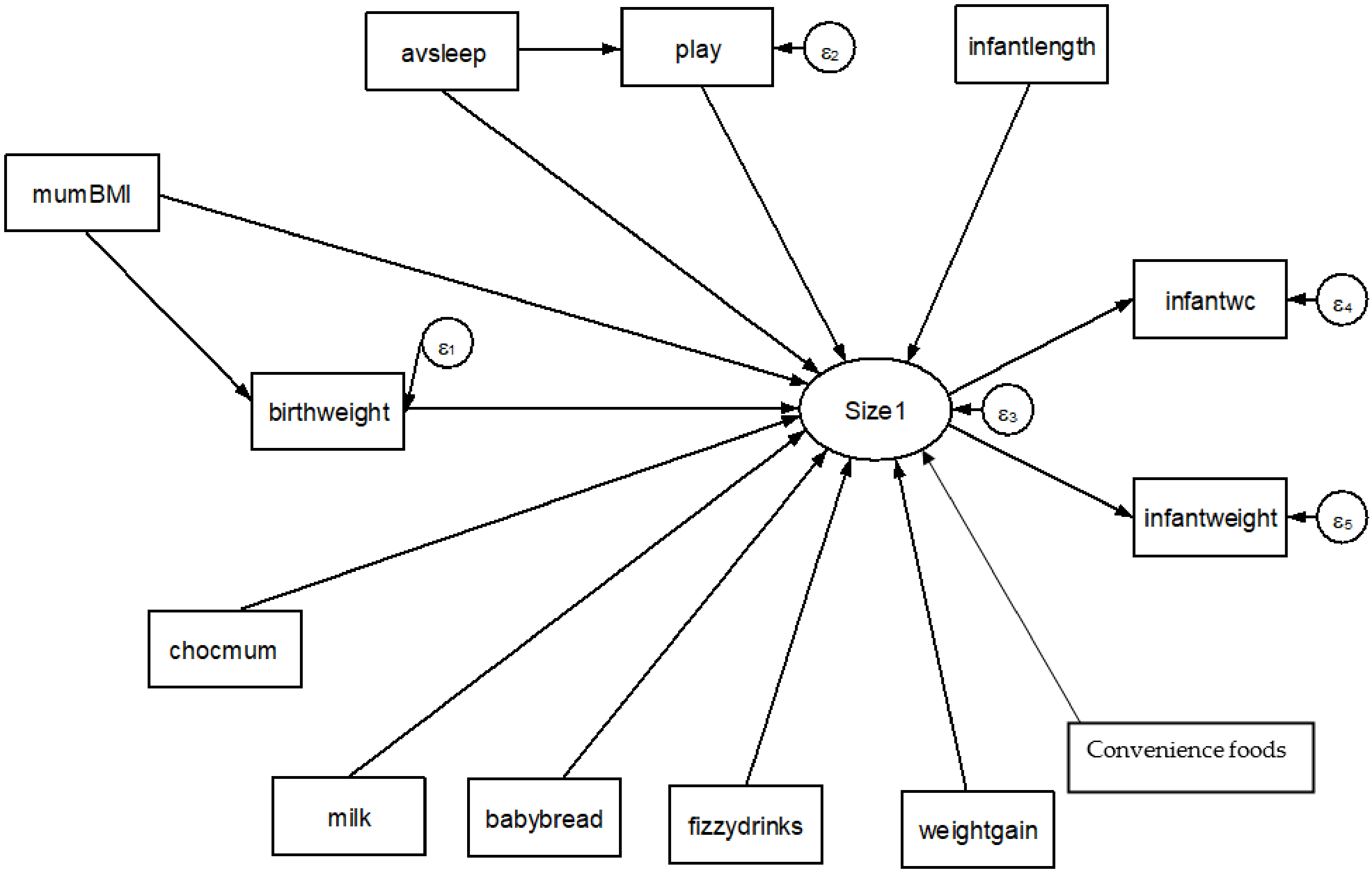

Figure S1. Exploratory structural equation model.

Method

Table S1. Study variables outlining details of assessment and time points of collection.

Variables	Measurement Unit	Source	Time Point
**Maternal exposures**			
Age at delivery	Infant date of birth—Mother date of birth	Questionnaire	Pregnancy
Ethnicity	White/non-white (0/1)	Questionnaire	Pregnancy
Pre-pregnancy BMI	kg/m^2	Medical records	Pregnancy
Skinkfold thickness	Sum of four skinfolds (mm); biceps, triceps, subscapular and suprailiac	Anthropometrist	Pregnancy and 12 m follow-up
Mid-arm circumference	cm	Anthropometrist	Pregnancy
Glucose	mmol/l	Anthropometrist	Pregnancy
Education level	University educated/A-levels /GCSE's /Trade /Other /None	Questionnaire	Pregnancy
Employment status	Full time/Part time/Seasonal/Homemaker/Student/ Unemployed	Questionnaire	Pregnancy and 12 m follow-up
Household Income	£10,000</£10,000–14,999/£15,000–24,999/£25,000–34,999/£35,000–39,999/£40,000–49,999/>£50,000	Questionnaire	Pregnancy and 12 m follow-up
Household allowance	£0/£50–149/£150–249/£250–499/£500–999/£1000+	Questionnaire	Pregnancy
Parity	0/1/2+	Medical records	Pregnancy
Depression score	Kessler 6 score where ≥12 signified	Questionnaire	Pregnancy
Smoking status	Yes or no (1/0)	Questionnaire	Pregnancy
Duration exposed to smoke	Daily/4–6 times per week/2–3 times a week/once a week/less often/not at all	Questionnaire	Pregnancy
Alcohol consumption	Yes or no (1/0)	Questionnaire	Pregnancy
Physical activity	Low activity/High activity (below/above 50th centile)	Accelerometer	Pregnancy
Daily consumption of carbohydrates	% of calories	7-day diet diary	Pregnancy
Daily consumption of total fat	% of calories	7-day diet diary	Pregnancy
Daily consumption of saturated fat	% of calories	7-day diet diary	Pregnancy
Daily consumption of protein	% of calories	7-day diet diary	Pregnancy
Daily consumption of fibre	Grams per day	7-day diet diary	Pregnancy
Alcohol consumption	Units per week	7-day diet diary	Pregnancy
Fruit consumption	Number per week	7-day diet diary	Pregnancy
Vegetable consumption	Number per week	7-day diet diary	Pregnancy
**Postnatal exposures**			
Birth weight	kg	Medical records	Birth
Head circumference	cm	Medical records	Birth
Apgar score	Reading at 1 and 5 minutes (scale 1–10)	Medical records	Birth
Growth rate per week	(Weight at approximately 6 months—birth weight)/24, above or below 0.16 g gain per week (0/1)	Medical records	6 months
Crawling milestone	Age at which first crawled (months)	Questionnaire	12 m follow-up
Walking at age 12 months	Yes/no (1/0)	Questionnaire	12 m follow-up
Breastfed	Yes/no (1/0)	Questionnaire	12 m follow-up

Duration of breastfeeding	Months	Questionnaire	12 m follow-up
Age first fed formula	Months	Questionnaire	12 m follow-up
Age first given solid foods	Months	Questionnaire	12 m follow-up
Milk given in past week	Only breast/only formula/ breast and formula/cow's milk/other	Questionnaire	12 m follow-up
Main type of food eaten	Homemade/readymade/take away (0/1/2)	Questionnaire	12 m follow-up
**Infant food frequency**			
Fruit consumption	Never/1–2/3–4/5–6/7 days per week	Questionnaire	12 m follow-up
Vegetable consumption	Never/1–2/3–4/5–6/7 days per week	Questionnaire	12 m follow-up
Bread/rice/pasta consumption	Never/1–2/3–4/5–6/7 days per week	Questionnaire	12 m follow-up
Fried food consumption	Never/1–2/3–4/5–6/7 days per week	Questionnaire	12 m follow-up
Take-away food consumption	Never/1–2/3–4/5–6/7 days per week	Questionnaire	12 m follow-up
Crisp consumption	Never/1–2/3–4/5–6/7 days per week	Questionnaire	12 m follow-up
Biscuits and cake consumption	Never/1–2/3–4/5–6/7 days per week	Questionnaire	12 m follow-up
Chocolate or sweet consumption	Never/1–2/3–4/5–6/7 days per week	Questionnaire	12 m follow-up
Carbonated drink consumption	Never/1–2/3–4/5–6/7 days per week	Questionnaire	12 m follow-up
**Infant sleep**			
Night time sleep	Hours and minutes (between 9 pm and 7 am)	Questionnaire	12 m follow-up
Day time sleep	Hours and minutes (between 7 am and 7 pm)	Questionnaire	12 m follow-up
Number of awakenings per night	0/1/2/3+	Questionnaire	12 m follow-up
Time spent in wakefulness	Hours and minutes	Questionnaire	12 m follow-up
Time taken to sleep	Hours and minutes	Questionnaire	12 m follow-up
Time of night sleep occurs	Hour and minutes	Questionnaire	12 m follow-up
Age infant first slept all night	Months	Questionnaire	12 m follow-up
Infant TV viewing duration	Hours (0/<1/1–2/3–4/5–6/6–7/8–9/10+)	Questionnaire	12 m follow-up
Maternal TV viewing duration	Hours (0/<1/1–2/3–4/5–6/6–7/8–9/10+)	Questionnaire	12 m follow-up
**12 m physical activity**			
Duration of readings	Number of days	Accelerometer	12 m follow-up
Time spent sedentary	Average number of hours	Accelerometer	12 m follow-up
Time spent active	Average number of hours	Accelerometer	12 m follow-up
**12 m anthropometrics**			
Length	cm	Anthropometrist	12 m follow-up
Weight	Kg	Anthropometrist	12 m follow-up
Mid-arm circumference	cm	Anthropometrist	12 m follow-up
Waist circumference	cm	Anthropometrist	12 m follow-up
Skinfold thicknesses	Sum of four skinfolds (mm); biceps, triceps, subscapular and suprailiac	Anthropometrist	12 m follow-up

Statistical Analysis

Table S2. Model Specification: Direct effect estimates for all variables with latent outcome—infant size.

Explanatory Variable	Number (%)	Mean (SD)	Slope (95% CI)	Mean Difference (95% CI)
**Demographics during pregnancy**				
Maternal age at delivery (years) (n = 218)				
18–25	39 (17.8)	-0.03 (0.8)	-	
26–30	64 (29.4)	-0.03 (0.8)	-0.06 (-0.3 to 0.2)	
31–35	63 (28.9)	-0.07 (0.8)	-0.17 (-0.5 to 0.1)	
≥36	52 (23.9)	-0.02 (0.8)	-0.01 (-0.3 to 0.3)	
Maternal age (continuous)	218			0.00 (-0.01 to 0.02)
Ethnicity (n = 225)				
White	186 (82.7)	0.02 (0.7)		-
Non-white	39 (17.3)	-0.10 (0.8)		0.11 (-0.2 to 0.4)
Mother education (n = 162)				
None	14 (8.6)	-0.44 (0.5)	-	
Trade or other	16 (9.9)	-0.12 (0.7)	0.15 (-0.6 to 0.9)	
School attainment	30 (18.5)	-0.10 (0.9)	0.38 (-0.2 to 1.0)	
University attainment	102 (63)	0.04 (0.7)	0.44 (-0.9 to 1.0)	
Father education (n = 141)				
None	8 (5.7)	-0.18 (0.7)	-	
Trade or other	37 (26.3)	0.21 (0.7)	0.56 (-0.2 to 1.3)	
School attainment	16 (11.3)	0.25 (0.9)	0.64 (-0.2 to 1.5)	
University attainment	80 (56.7)	-0.10 (0.7)	-0.23 (-0.5 to 0.9)	
Mother employment (n = 218)				
Unemployed/Homemaker	54 (24.8)	-0.18 (0.8)		-
Employed	164 (75.2)	0.03 (0.7)		-0.21 (-0.4 to 0.01)
Father employment (n = 200)				
Unemployed	18 (9)	0.06 (0.6)		
Employed	182 (91)	0.00 (0.7)		0.1 (-0.3 to 0.4)
Household allowance (£) (n = 225)				
£0	74 (32.9)	-0.02 (0.7)	-	
£50–250	61 (27.1)	0.19 (0.7)	0.21 (-0.03 to 0.5)	
£251–1000	90 (40.0)	-0.12 (0.8)	-0.10 (-0.43to 0.1)	
**Maternal lifestyle during pregnancy**				
Passive smoking (n = 225)				
No	146 (64.9)	0.07 (0.7)		-
Yes	79 (35.1)	-0.13 (0.8)		0.20 (-0.00 to 0.4)
Smoking in pregnancy				
No	176 (84.2)	-0.003 (0.7)		-
Yes	35 (15.8)	0.002 (0.8)		0.01 (-0.3 to 0.3)
Parity (n = 225)				
≤1	187 (83.1)	0.03 (0.7)		
>1	38 (16.9)	-0.15 (0.8)		0.18 (-0.1 to 0.4)
**Maternal activity**				
Maternal exercise (bouts per week) (n = 151)				
0	57 (37.7)	-0.04 (0.7)	-	
1	36 (23.8)	0.16 (0.7)	0.20 (-0.1 to 0.5)	
2 to 3	41 (27.2)	0.01 (0.8)	0.05 (-0.2 to 0.3)	
>3	17 (11.3)	0.3 (0.6)	0.31 (-0.1 to 0.7)	
**Maternal dietary consumption (n = 158)**				
Recommended fat (%)				
Yes	94 (59.5)	0.03 (0.7)		-
No	64 (40.5)	0.06 (0.8)		-0.03 (-0.3 to 0.2)
Recommended saturated fat (%)				
Yes	37 (23.4)	-0.03 (0.6)		-
No	121 (76.6)	0.06 (0.8)		-0.09 (-0.4 to 0.2)

Recommended carbohydrate (%)				
Yes	127 (80.4)	0.04 (0.7)		-
No	31 (19.6)	0.03 (0.8)		0.02 (-0.3 to 0.3)
Maternal fruit (portions)				
0	21 (13.3)	0.02 (0.9)	-	
1–10	78 (49.4)	0.11 (0.7)	0.09 (-0.2 to 0.4)	
1120	47 (29.7)	-0.13 (0.7)	-0.15 (-0.5 to 0.2)	
20+	12 (7.6)	0.26 (0.6)	0.24 (-0.3 to 0.8)	
Maternal vegetable (portions)				
0–10	95 (60.1)		-	
11–20	51 (32.3)		-0.06 (-0.3 to 0.2)	
20+	12 (7.6)		-0.03 (-0.5 to 0.4)	
**Perinatal outcomes**				
Gender (n = 125)				
Female	103 (47.4)	-0.19 (0.7)		
Male	122 (52.6)	0.16 (0.7)		0.35 (0.2 to 0.5)
Gestation (weeks) (n = 225)				
<37	8 (3.6)	-0.24 (0.8)		-
≥37	217 (96.4)	0.01 (0.7)		-0.25 (-0.8 to 0.3)
Gestation (Continuous)			0.01 (-0.1 to 0.1)	
Apgar 1 minutes (n = 225)				
<7	8 (3.6)	-0.12 0.7)		-
≥7	217 (96.4)	0.00 (0.7)		-0.12 (-0.6 to 0.4)
Induction (n = 91)				
Yes	21 (23.1)	0.24 (0.7)		-
No	70 (76.9)	-0.00 (0.7)		-0.24 (-0.6 to 0.1)
Smoking at birth (n = 84)				
Yes	9 (10.7	-0.23 (0.6)		-
No	75 (89.3)	0.08 (0.7)		0.31 (-0.2 to 0.8)
Siblings born macrosomic (n = 100)				
No	85 (85)	-0.07 (0.7)		
Yes	15 (15)	0.37 (0.8)		-0.44 (-0.9 to -0.03)
**Demographics at 12 months**				
Mother employment (n = 223)				
Unemployed	51 (22.9)	-0.08 (0.8)		-
Employed	172 (77.1)	-0.02 (0.7)		-0.06 (-0.3 to 0.2)
Father employment (n = 187)				
Unemployed	5 (3.2)	0.22 (0.4)		-
Employed	182 (96.8)	-0.04 (0.7)		0.26 (-0.4 to 0.9)
Household income (n = 175)				
£0–15,000	38 (21.7)	-0.10 (0.7)		-
£15,000+	137 (78.3)	0.06 (0.7)		-0.16 (-0.4 to 0.1)
Reported financial state (n = 193)				
Comfortable	37 (19.1)	-0.01 (0.6)	-	
Doing OK	81 (42)	-0.02 (0.8)	0.07 (-0.2 to 0.4)	
Just getting by	54 (28)	0.09 (0.7)	0.17 (-0.1 to 0.5)	
Quite difficult	17 (8.8)	-0.31 (0.9)	-0.22 (-0.6 to 0.2)	
Very difficult	4 (2.1)	0.19 (0.7)	0.27 (-0.5 to 1.1)	
**Maternal anthropometrics at 12 m**				
Total skinfold thickness (n = 225)				
Low tertile (<60.6 mm)	78 (34.6)	-0.11 (0.7)	-	
Middle tertile (60.6–104 mm)	68 (30.3)	-0.04 (0.9)	0.08 (-0.2 to 0.3)	
High tertile (>104 mm)	79 (35.1)	0.14 (0.7)	0.26 (0.02 to 0.5)	
Total skinfold thickness (Continuous)			0.002 (-.00 to 0.01)	
**Maternal lifestyle at 12 m**				
TV viewing per day (n = 193)				
0–1 hour	28 (14.5)	0.12 (0.7)	-	

1–2 hours	81 (42)	-0.04 (0.7)	-0.17 (-0.5 to 0.1)	
3–4 hours	58 (30.1)	-0.03 (0.7)	-0.16 (-0.5 to 0.2)	
5+ hours	26 (13.4)	0.10 (0.8)	-0.22 (-0.6 to 0.2)	
Depression score (n = 194)				
<12	188 (95.9)	-0.02 (0.7)		-
≥12	6 (4.1)	-0.11 (0.9)		0.09 (-0.5 to 0.7)
Maternal exercise (bouts per week) (n = 105)				
0	36 (34.3)	-0.09 (0.8)	-	
1	21 (20)	-0.09 (0.7)	-0.08 (-0.4 to 0.5)	
2 to 3	28 (26.7)	0.20 (0.8)	0.30 (-0.1 to 0.7)	
>3	20 (19.0)	0.06 (0.7)	0.15 (-0.3 to 0.6)	
**Maternal food frequency (days of consumption) (n = 190)**				
Fruit				
7	83 (43.7)	0.08 (0.7)	-	
5–6	33 (17.4)	0.04 (0.7)	-0.04 (-0.3 to 0.3)	
3–4	38 (20)	-0.10 (0.7)	-0.18 (-0.5 to 0.1)	
2 or less	36 (18.9)	-0.18 (0.8)	-0.26 (-0.5 to 0.03)	
Vegetables				
7	108 (56.8)	0.06 (0.7)	-	
5-6	36 (18.9)	-0.07 (0.7)	-0.13 (-0.4 to 0.1)	
4 or less	46 (24.2)	-0.13 (0.7)	-0.18 (-0.5 to 0.1)	
Bread, rice, pasta or potatoes				
7	131 (68.9)	0.02 (0.7)	-	
5–6	30 (15.8)	-0.06 (0.7)	-0.09 (-0.4 to 0.2)	
4 or less	29 (15.3)	-0.15 (0.7)	-0.17 (-0.5 to 0.1)	
Fried foods (n = 222)				
4 or more	30 (16)	-0.01 (0.8)	-	
1–2	100 (53.5)	0.02 (0.7)	0.03 (-0.3 to 0.3)	
Never	57 (30.5)	-0.08 (0.7)	-0.06 (-0.4 to 0.3)	
Take-away foods (n = 220)				
1 or more	132 (70.6)	-0.02 (-07)		
Never	55 (29.4)	0.01 (0.8)		-0.13 (-0.4 to 0.2)
Convenience foods (n = 184)				
1 or more	78 (42.4)	-0.06 (0.7)		
Never	106 (57.6)	0.02 (0.7)		-0.08 (-0.3 to 0.1)
Crisps (n = 223)				
3–4	51 (27.1)	-0.14 (0.7)	-	
1–2	75 (39.9)	0.09 (0.7)	0.23 (-0.03 to 0.4)	
Never	62 (33)	-0.04 (0.8)	0.10 (-0.2 to 0.4)	
Biscuits (n = 189)				
1–7	161 (85.2)	0.01 (0.1)		-
Never	28 (14.8)	-0.14 (0.1)		-0.15 (-0.5 to 0.1)
Chocolate (n = 187)				
1 or more	150 (80.2)	0.05		-
Never	37 (19.8)	-0.27 (0.7)		0.32 (0.07 to 0.6)
Carbonated drinks				
3–4	60 (31.9)	-0.07 (0.8)		
1–2	57 (30.3)	0.05 (0.7)	0.12 (-0.2 to 0.4)	
Never	71 (37.8)	-0.01 (0.7)	0.06 (-0.2 to 0.3)	
**Infant lifestyle**				
Weight gain per week (birth to 6 months) (n = 225)				
low tertile (≤0.16 g)	115 (51.1)	-0.11 (0.7)	-	
high tertile (>0.16 g)	110 (48.9)	0.11 (0.8)	-0.22 (-0.4 to -0.03)	
Weight gain per week (Continuous)			4.0 (1.4 to 6.5)	
**Sleep**				
Wakings per night (n = 225)				

<2	129 (57.3)	0.01 (0.7)	-0.14 (-0.5 to 0.2)	
≥2	96 (42.7)	-0.01 (0.8)	-0.07 (-0.4 to 0.2)	
Wakings per night (Continuous)			-0.07 (-0.1 to 0.0)	
Mean daily sleep duration (hours per day) (n = 216)				
<10	15 (6.9)	-0.44 (0.6)	-	
10–12	68 (31.5)	0.07 (0.7)	0.51 (0.1 to 0.9)	
12.5–14	81 (37.5)	-0.01 (0.7)	0.43 (0.3 to 0.8)	
15–17	52 (24.1)	0.10 (0.7)	0.54 (0.1 to 0.9)	
Mean daily sleep duration (Continuous)			0.11 (0.04 to 0.2)	
Age first slept through (n = 219)				
0–3 months	112 (51.4)	-0.06 (0.7)	-	
>3–6 months	56 (25.6)	0.05 (0.7)	0.12 (-0.1 to 0.4)	
>6–12 months	45 (20.6)	0.13 (0.7)	0.20 (-0.1 to 0.5)	
Never	6 (2.7)	-0.82 (0.6)	-0.75 (-1.4 to -0.1)	
Age first slept through (Continuous)			-0.01 (-0.01 to -0.001)	
Sleep considered a problem (n = 192)				
No	48 (25)	-0.02 (0.7)		-
Yes	144 (75)	-0.01 (0.7)		-0.004 (-0.2 to 0.2)
**Infant development**				
Walking at age 12 months (n = 194)				
Yes	103 (53.1)	-0.04 (0.7)		-
No	91 (46.9)	0.00 (0.8)		-0.04 (-0.2 to 1.2)
Age first crawled (n = 187)				
≤6 months	42 (22.5)	-0.09 (0.6)	-	
>6–9 months	95 (50.8)	-0.02 (0.7)	0.07 (-0.2 to 0.6)	
9–12 months	41 (21.9)	0.04 (0.8)	0.12 (-0.2 to 0.7)	
Never	9 (4.8)	0.07 (0.8)	0.16 (-0.4 to 0.9)	
**Infant activity**				
Play with infant (n = 194)				
More than once a day	190 (97.9)	-0.01 (0.7)		-
Less often	4 (2.1)	-0.69 (0.6)		0.68 (-0.04 to 1.4)
Play physically active games (n = 190)				
Every day	156 (82.1)	0.06 (0.7)		-
Less often	34 (17.9)	-0.32 (0.7)		-0.36 (-0.7 to 0.0)
Frequency going to park (n = 194)				
Every day	12 (6.2)	-0.21 (0.6)	-	
Several times a week	136 (70.1)	0.00 (0.8)	0.22 (-0.2 to 0.7)	
Monthly/Never	46 (23.7)	-0.04 (0.7)	0.17 (-0.3 to 0.6)	
TV viewing per day (n = 193)				
None	38 (19.7)	-0.09 (0.7)	-	
less than 1 hour	68 (35.2)	0.06 (0.7)	0.15 (-0.1 to 0.4)	
1–2 hours	52 (26.9)	-0.10 (0.8)	-0.02 (-0.3 to 0.3)	
3+ hours	35 (18.1)	0.01 (0.7)	0.09 (-0.2 to 0.4)	
Time spent sedentary (hours) (n = 44)				
<5	12	0.42 (0.1)		-
5+	32	-0.11 (0.1)		0.53 (0.07 to 0.98)
Time spent sedentary (Continuous)			-0.03 (-0.1 to 0.0)	
Intensity of time spent active (SVM)				
Low tertile (<175832.1)	19	-0.02 (0.7)	-	
Middle tertile (175832.1)	18	0.17 (0.6)	0.18 (-0.2 to 0.6)	
high tertile (302475.5)	16	-0.16 (0.5)	-0.14 (-0.6 to 0.3)	
**Infant feeding**				
Initial mode of feeding (n = 194)				
Breastfed	165 (85.1)	-0.02 (0.7)		-
Formula	29 (14.9)	-0.02 (0.8)		-0.00 (-0.3 to 0.3)
Mode of feeding at 12 months (n = 193)				

Breast	25 (13)	-0.31 (0.6)		-
Other milk	168 (87)	0.03 (0.7)		-0.35 (-0.7 to -0.1)
Duration of breastfeeding (n = 225)				
<6 months	94 (41.8)	-0.09 (0.8)		-
6+ months	131 (58.2)	0.06 (0.7)		-0.15 (-0.3 to 0.04)
Introduction of solids (months) (n = 189)				
<4	56 (29.6)	0.06 (0.8)		-
4+	133 (70.4)	-0.06 (0.7)		0.12 (-0.1 to 0.4)
Age of introduction (Continuous)			-0.03 (-0.1 to 0.04)	
**Infant food frequency (days of consumption)**				
Vegetables (n = 193)				
7	144 (74.6)	-0.01 (0.8)	-	
5–6	20 (10.4)	-0.04 (0.6)	-0.04 (-0.4 to 0.4)	
0–4	29 (15)	-0.09 (0.7)	-0.19 (-0.4 to 0.2)	
Bread, rice, pasta or potatoes (n = 191)				
7	138 (70.1)	0.07 (0.1)		-
Less than 7	53 (11.7)	-0.21 (0.1)		-0.28 (-0.5 to -0.1)
Fried foods (n = 188)				
3–7	12 (6.4)	-0.09 (0.8)	-	
1–2	45 (23.9)	-0.04 (0.8)	0.05 (-0.4 to 0.5)	
Never	131 (69.7)	0.02 (0.7)	0.11 (-0.3 to 0.5)	
Take-away foods (n = 186)				
1–7	22 (10.5)	0.11 (0.8)		-
Never	164 (89.5)	-0.01 (0.7)		-0.12 (-0.4 to 0.2)
Convenience foods (n = 188)				
3–7	48 (25.5)	-0.18 (0.1)		-
0–2	140 (74.5)	0.06 (0.1)		0.24 (0.01 to 0.5)
Crisps (n = 190)				
5–7	14 (7.4)	-0.26 (0.8)	-	
3–4	22 (11.6)	0.05 (0.8)	0.31 (-0.2 to 0.8)	
1-2	58 (30.5)	0.06 (0.8)	0.33 (-0.1 to 0.7)	
Never	96 (50.5)	-0.02 (0.7)	0.24 (-0.2 to 0.6)	
Biscuits or cakes (n = 190)				
5–7	22 (11.6)	-0.18 (0.8)	-	
3–4	37 (19.5)	0.09 (0.8)	0.28 (-0.1 to 0.7)	
1–2	85 (44.7)	0.04 (0.7)	0.22 (-0.1 to 0.5)	
Never	46 (24.2)	-0.08 (0.6)	0.10 (-0.3 to 0.5)	
Chocolate or sweets (n = 192)				
3–7	18 (9.4)	-0.13 (0.7)	-	
1–2	61 (31.8)	-0.10 (0.9)	0.03 (-0.4 to 0.4)	
Never	113 (58.8)	0.06 (0.6)	0.19 (-0.2 to 0.6)	
Carbonated drinks (n = 191)				
1–7	11 (5.8)	-0.52 (0.8)		-
Never	180 (94.2)	0.03 (0.7)		0.55 (0.1 to 0.9)

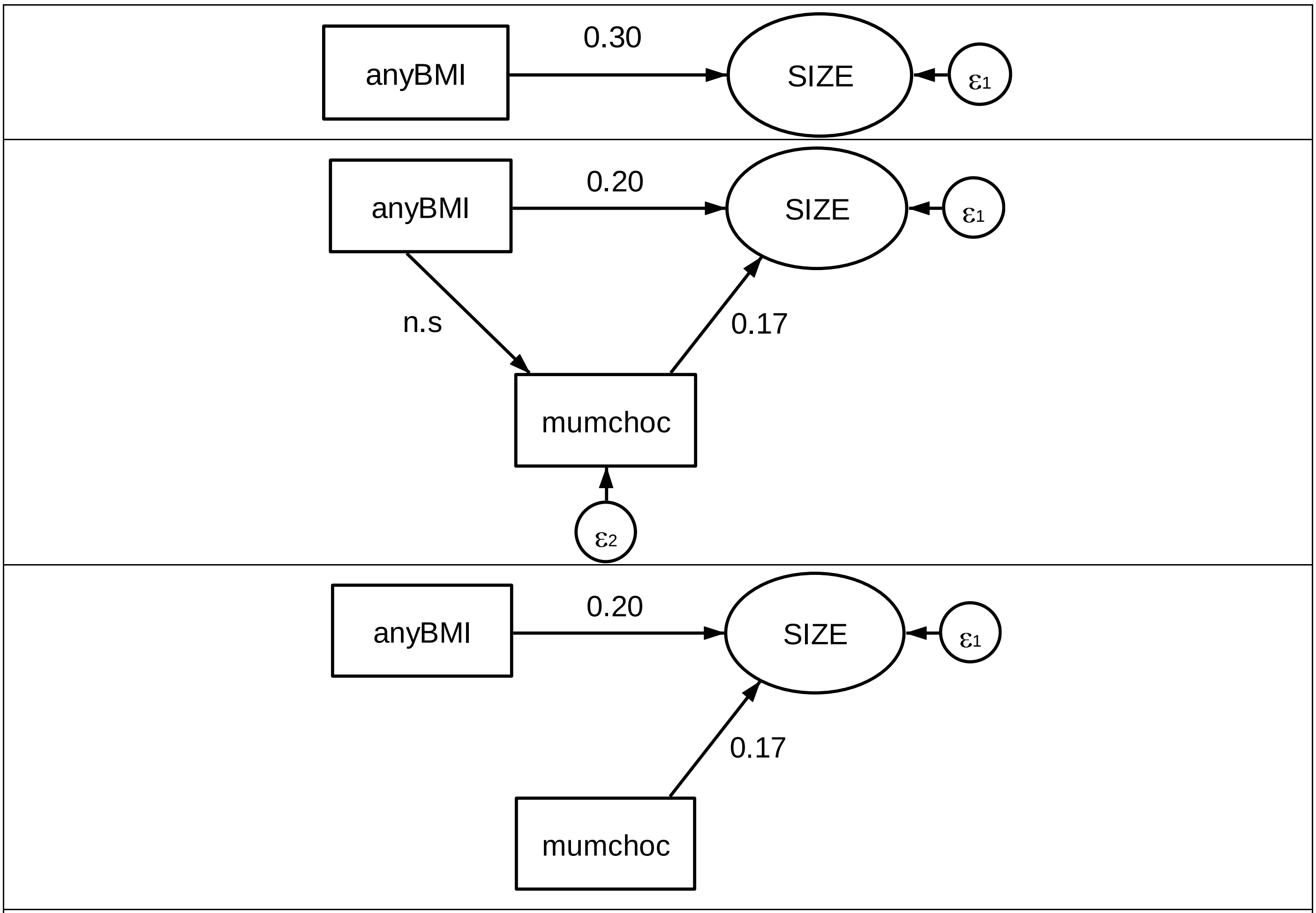
anyBMI
0.30
SIZE
ε1
anyBMI
0.20
SIZE
ε1
n.s
0.17
mumchoc
ε2
anyBMI
0.20
SIZE
ε1
0.17
mumchoc

(**a**)

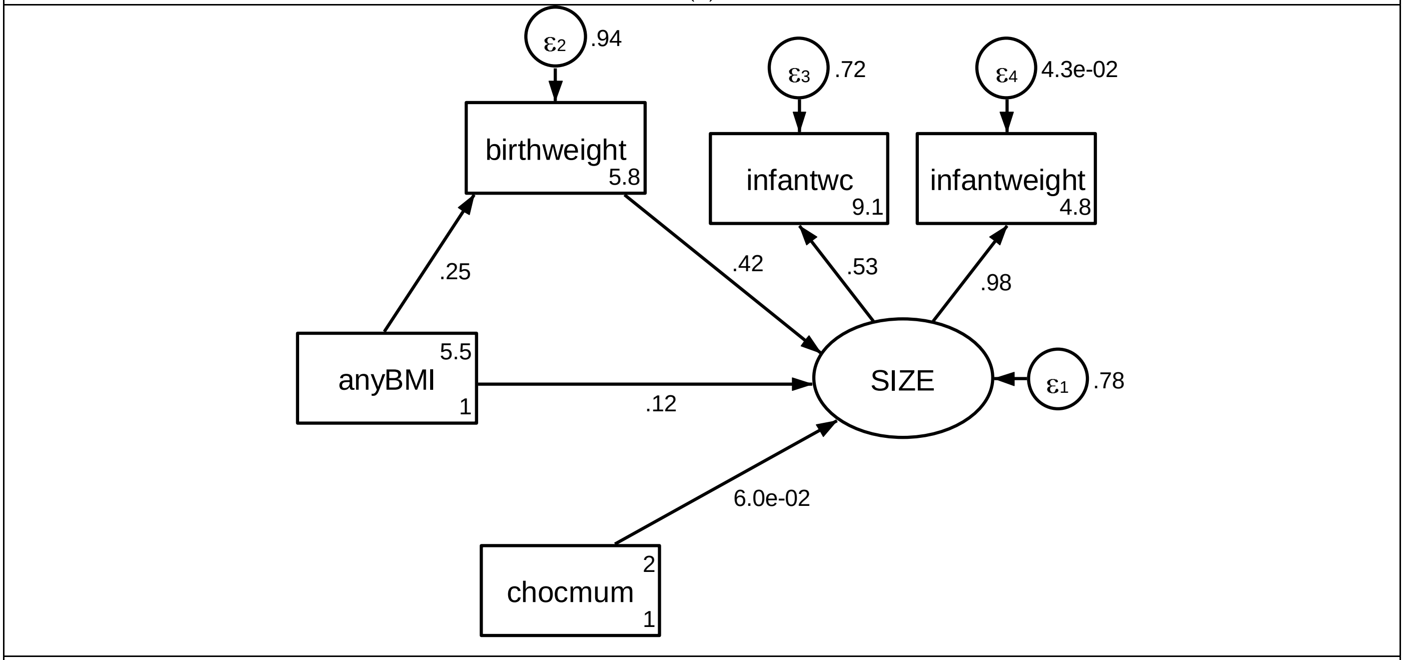
ε2 .94
ε3 .72
ε4 4.3e-02
birthweight
5.8
infantwc
9.1
infantweight
4.8
.25
.42
.53
.98
5.5
anyBMI
1
SIZE
ε1 .78
.12
6.0e-02
2
chocmum
1

(**b**)

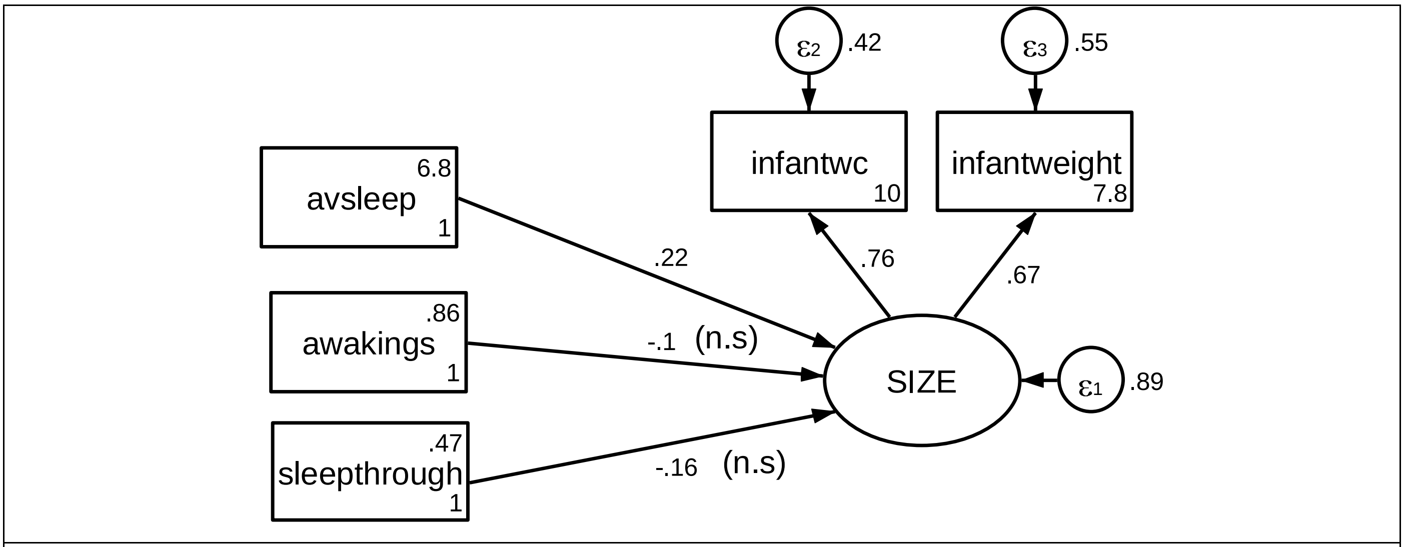

ε2 .42
ε3 .55
infantwc
10
infantweight
7.8
avsleep
6.8
1
.22
.76
.67
awakings
.86
1
-.1 (n.s)
SIZE
ε1 .89
sleepthrough
.47
1
-.16 (n.s)

(c)

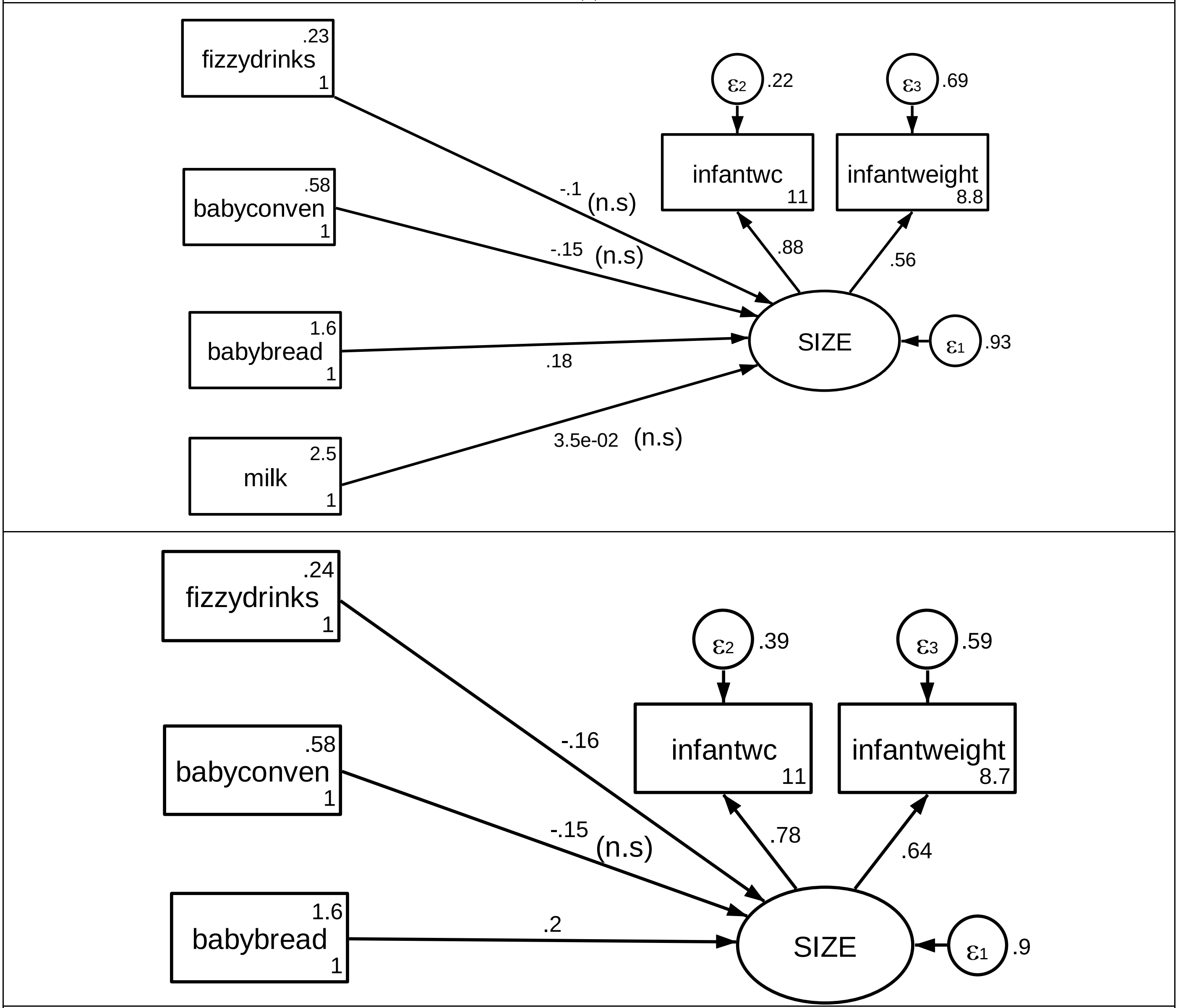

fizzydrinks
.23
1
ε2 .22
ε3 .69
infantwc
11
infantweight
8.8
babyconven
.58
1
-.1 (n.s)
-.15 (n.s)
.88
.56
babybread
1.6
1
.18
SIZE
ε1 .93
milk
2.5
1
3.5e-02 (n.s)
fizzydrinks
.24
1
ε2 .39
ε3 .59
infantwc
11
infantweight
8.7
babyconven
.58
1
-.16
-.15 (n.s)
.78
.64
babybread
1.6
1
.2
SIZE
ε1 .9

(d)

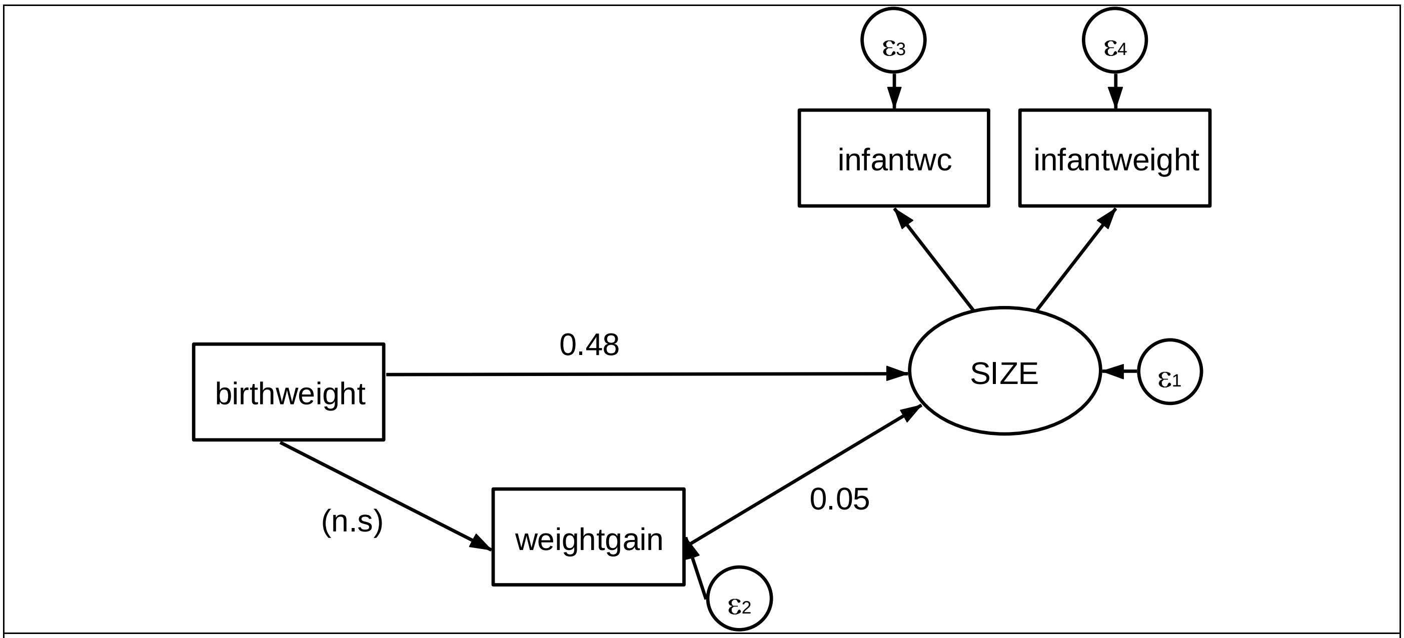

(**e**)

Figure S2. Identifying pathways within the exploratory SEM. Note: All findings are reported as standardised coefficients. (**a**) Step 1: Testing the relation between maternal factors. (**b**) Step 2: Consideration of infant birth weight. (**c**) Step 3: Inclusion of infant sleep factors. (**d**) Step 4: Inclusion of infant feeding factors. Infant milk was removed from the model as it revealed the lowest significant association ($p > 0.1$), thereafter infant consumption of convenience foods was removed ($p > 0.1$). (**e**) Step 5: Infant birth weight and weight gain relations

Key to figures

Symbol/Label	Meaning
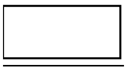	Observed Variable
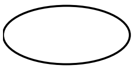	Latent variable
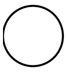	Residual error
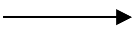	Predictive relationship/hypothesised pathway
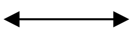	Correlation
(n.s)	Non-significant association
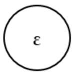	Error terms i.e., residual variances
anyBMI	Pre-pregnancy BMI
Mumchoc	Maternal chocolate consumption at 12 months
Infantwc	Infant waist circumference
Babybread	Infant carbohydrate consumption
Babyconven	Infant consumption of convenience foods
Fizzydrinks	Infant consumption of carbonated drinks
Milk	Consumption of milk (other than breast milk) at age 12 months
Avsleep	Average length of infant sleep
Awakings	Number of times infant awakes per night

Sleepthrough	Age infant first slept all night
Weightgain	Average infant weight gain per week (from 0–6 months of age)

Table S3. Descriptive statistics for all variables included within the modified model.

Construct and Related Variables	Values
Pre-pregnancy BMI	
Body mass index at 12 weeks gestation (in kg/m^2)	24.7 ± 4.7 (16.7–54)
Birth weight	
Kilograms (kg)	3.44 ± 0.49 (1.75–4.54)
Infant play	
Questionnaire	0.18 ± 0.4
Infant carbohydrate consumption	
Food frequency questionnaire	0.72 ± 0.5
Infant fizzy drink consumption	
Food frequency questionnaire	0.06 ± 0.23
Infant weight gain	
Average weekly weight gain from birth to 6 months (g)	0.14 ± 0.04 (0.02–0.26)
Infant length	
Measured at age 12 months (cm)	76.85 ± 5.1 (46.7–98)
Infant size	
Infant waist circumference at age 12 months (cm)	46.71 ± 4.1 (32.2–56.1)
Infant weight at age 12 months (kg)	9.80 ± 1.2 (6.7–13.3)

Mean ± SD with range in parentheses.

Discussion

Data availability affected our capacity to include potentially important variables within our model. Recognising the importance of body composition when evaluating infant size, the present study gathered skinfold thickness measures at age 1 year. As a child's BMI does not remain relatively constant with a dramatic rise in fat mass throughout the first year of life, it is desirable to distinguish between fat mass and fat-free mass. Unfortunately, due to insufficient data, inclusion of this variable within our SEM analyses was not possible. Reasons for the unavailability of data include; difficulty in obtaining measures in individuals at a young age, consent not given to gather the measures and no follow-up visit data. Once the cohort has achieved a higher number of participants, future work shall look to evaluate determinants of infant body composition.

We were also unable to include objective measures of physical activity within our model. Despite preliminary analyses revealing a significant association between the time infant's spent sedentary and their size at age 12 months, a lack of data prevented its consideration. We did however include a proxy variable; time spent playing physically active games, reported by the mother in a questionnaire.
